# Drug utilization, prescription errors and potential drug-drug interactions: an experience in rural Sri Lanka

**DOI:** 10.1186/s40360-016-0071-z

**Published:** 2016-06-25

**Authors:** Devarajan Rathish, Sivaswamy Bahini, Thanikai Sivakumar, Thilani Thiranagama, Tharmarajah Abarajithan, Buddhika Wijerathne, Channa Jayasumana, Sisira Siribaddana

**Affiliations:** Department of Pharmacology, Faculty of Medicine and Allied Sciences, Rajarata University of Sri Lanka, Saliyapura, Sri Lanka; Department of Physiology, Faculty of Medicine and Allied Sciences, Rajarata University of Sri Lanka, Saliyapura, Sri Lanka; Department of Forensic Medicine, Faculty of Medicine and Allied Sciences, Rajarata University of Sri Lanka, Saliyapura, Sri Lanka; Department of Medicine, Faculty of Medicine and Allied Sciences, Rajarata University of Sri Lanka, Saliyapura, Sri Lanka

**Keywords:** Drug utilization, Prescription error, Prescription analysis, Drug interactions, Sri Lanka

## Abstract

**Background:**

Prescription writing is a process which transfers the therapeutic message from the prescriber to the patient through the pharmacist. Prescribing errors, drug duplication and potential drug-drug interactions (pDDI) in prescriptions lead to medication error. Assessment of the above was made in prescriptions dispensed at State Pharmaceutical Corporation (SPC), Anuradhapura, Sri Lanka.

**Methods:**

A cross sectional study was conducted. Drugs were classified according to the WHO anatomical, therapeutic chemical classification system. A three point Likert scale, a checklist and Medscape online drug interaction checker were used to assess legibility, completeness and pDDIs respectively.

**Results:**

Thousand prescriptions were collected. Majority were hand written (99.8 %) and from the private sector (73 %). The most frequently prescribed substance and subgroup were atorvastatin (4 %, *n* = 3668) and proton pump inhibitors (7 %, *n* = 3668) respectively. Out of the substances prescribed from the government and private sectors, 59 and 50 % respectively were available in the national list of essential medicines, Sri Lanka. Patients address (5 %), Sri Lanka Medical Council (SLMC) registration number (35 %), route (7 %), generic name (16 %), treatment symbol (48 %), diagnosis (41 %) and refill information (6 %) were seen in less than half of the prescriptions. Most were legible with effort (65 %) and illegibility was seen in 9 %. There was significant difference in omission and/or errors of generic name (*P* = 0.000), dose (*P* = 0.000), SLMC registration number (*P* = 0.000), and in evidence of pDDI (*P* = 0.009) with regards to the sector of prescribing. The commonest subgroup involved in duplication was non-steroidal anti-inflammatory drugs (NSAIDs) (43 %; 56/130). There were 1376 potential drug interactions (466/887 prescriptions). Most common pair causing pDDI was aspirin with losartan (4 %, *n* = 1376).

**Conclusion:**

Atorvastatin was the most frequently prescribed substance. Fifteen percent of the prescriptions originate from government sector. SLMC registration number and trade names were seen more in prescriptions originating from the private sector. Most prescriptions were legible with effort. NSAIDs were the commonest implicated in drug class duplication. Fifty three percent of prescriptions have pDDI.

**Electronic supplementary material:**

The online version of this article (doi:10.1186/s40360-016-0071-z) contains supplementary material, which is available to authorized users.

## Background

Prescription writing is a science, art and a basic skill that every prescriber needs to learn. It transfers the therapeutic message from the prescriber to the patient through the pharmacist [1]. An error in a prescription may introduce adverse effects and potential drug - drug interaction (pDDI). Drug related complications were found to be common in hospital based studies [2]. Guidance on prescribing is provided in the British National Formulary (BNF) and in a practical manual published by World Health Organization (WHO) [3, 4].

Knowledge on drug utilization in a particular population can be used to improve the supply of drugs to that particular population. The present drug dispensing system in Sri Lanka makes it difficult to assess drug utilization. Reasons are poor record keeping, lack of coordination between government and private sector and drug dispensing without, expired or incorrect prescriptions.

The prescribing error is defined as follows

“A clinically meaningful prescribing error occurs when, as a result of a prescribing decision or prescription writing process, there is an unintentional but significant

i. Reduction in the probability of treatment being timely and effective or
ii. Increase in the risk of harm when compared with generally accepted practice” [5].

Legibility of prescriptions is a legal duty of the prescriber. The traditional prescription writing by hand inherits high incidence of documentation errors [6]. There are instance where the prescriber has been found guilty for the death of a patient as a result of illegible prescription [7]. Previous studies have revealed the prevalence of prescribing errors in prescriptions [8].

Drug interactions lead to toxicity or therapeutic failure [9]. Resources to check pDDIs include formularies, interaction tables, prescribing & dispensing software and drug information services (e.g.,; Stockley’s Drug Interactions, Medscape, Micromedex) [9].

The Sri Lankan health system includes allopathic, ayurvedic and Sinhala medicine. Allopathic system consist a universal free government sector and the fee levying private sector. Though Sri Lanka’s total expenditure on health is 3.2 % of gross domestic product (GDP) for the year 2013 its health indicators are comparable with many of the more developed countries in Asia [10, 11].

Anuradhapura is the largest district in Northcentral province and in Sri Lanka, with a population of nearly 856,500 by 2012 [12]. Majority of its population (94.6 %) belong to the rural sector [12] and agriculture (55 %) is their main employment (unemployment rate is 3.1 %) [13]. The mean monthly household income of Anuradhapura is Sri Lankan rupees 35,460, which is low compared to the overall mean monthly household income of the country (Sri Lankan rupees 45,878) [14]. The only tertiary care hospital available for the entire district is situated in Anuradhapura town, the teaching hospital Anuradhapura which is owned by the government. This makes it the only choice for patients of Anuradhapura to seek specialized care such as cardiology, intensive care, nephrology etc. The SPC of Sri Lanka is an institute that procures, produces and distributes pharmaceuticals with retail outlets Island wide. It was established in 1971 as a result of the work by Prof. Senaka Bibile and Dr. S.A. Wickramasinghe [15]. It promotes generic prescribing and sells drugs in a much affordable price compared to private pharmacies in the country. The only outlet of SPC in Anuradhapura is situated very closer to the teaching hospital Anuradhapura. The next outlet of SPC is either in Polonnaruwa, Kurunegala or Jaffna districts which are 100, 115 and 200 km away respectively [15]. Therefore large number of low income and rural population visits aforementioned SPC to obtain drugs.

In Sri Lankan health system, there are no regulations in practice to monitor drug prescription and to define the maximum number of drugs per prescription. This is a leeway for higher number of prescription errors and pDDIs per prescription.

Although there are few data from urban areas of Sri Lanka, studies on drug utilization and prescription errors of rural Sri Lanka are scarce. Knowledge on these would help identify and address issues related specifically to rural Sri Lanka. Our main objective was to assess prescriptions dispensed in rural region for drug utilization, prescription errors and pDDIs. The pDDI are due to errors in prescribing. pDDIs and prescription errors will indirectly dependent on patterns of drug utilization. Findings of the patterns of drug utilization, prescription errors and pDDIs are interrelated and cannot be segregated. This would help to address them subsequently. We chose State Pharmaceutical Corporation (SPC), Anuradhapura, Sri Lanka as our sample population.

## Methods

A descriptive cross sectional study was conducted at SPC, Anuradhapura, Sri Lanka from May to August 2015. Every third prescription received at the SPC Anuradhapura was selected. The WHO anatomical, therapeutic chemical (ATC) classification system was used to group the chemical substance. At the 1st level the drugs are divided into 14 groups based on the anatomical system they act on. Second level includes therapeutic subgroups. The 3rd and 4th levels are usually pharmacological and chemical subgroups. The 5th level is the chemical substance. The classification system uses International non-proprietary names. If this name is not assigned to a particular drug then the system uses United States adopted name or British approved name [16]. All the chemical substances were checked for inclusiveness in the national list of essential medicines in Sri Lanka [17].

The 3 points of the likert scale used to assess legibility (the quality of being clear enough to read) were as follows: 1 - Illegible, 2 - Legible with effort and 3 - Legible. The checklist (Additional file 1) used to assess completeness included components of the prescription mentioned both in the BNF and WHO practical manual [3, 4]. Completeness was defined for each component according to the above two resource materials. The components were:

- Patient information (name, age, gender and address)
- Prescriber information (name, signature, Sri Lanka Medical Council (SLMC) registration number below prescribers’ signature, place of prescribing, contact details, qualifications and prescriber's rubber stamp)
- Drug information {route of administration, generic name, dose, frequency, duration and information for the package label (the information written by the prescriber and copied by the pharmacist onto the label of the package. This includes the amount of drug to be taken, frequency, any specific instructions and warnings [4])}
- Other information (date & treatment symbol).

In addition, diagnosis of the disease and refill information was included [18–20]. Data was anonymized so identity of the patients or the prescribers was not collected. Both the assessment of legibility and filling of the checklist were done by trained Bachelor of Medicine, Bachelor of Surgery (MBBS) qualified doctors. MBBS is the first professional degree awarded, in medicine and surgery upon graduation from medical school, by universities in countries that follow the common wealth tradition. The tools were pre-tested for accuracy before the data collection proper.

Drug duplication was defined as duplication of same chemical substance in a single prescription. Drug class duplication was defined as duplication of same chemical sub-group in a single prescription.

The pDDIs were defined as each pair of drugs known for possible interactions. When a single drug combination caused pDDIs through more than one mechanism, the mechanism of the most severe pDDIs was considered. Medscape drug interaction checker was used to identify pDDIs. It is an open access information service which verifies interactions between brand and generic drugs, over-the-counter drugs and supplements [21]. Up to 30 drugs, herbals, and supplements can be checked at a time. The software is available online, as a mobile application and the results can be printed. There are three types of interactions, serious, significant and minor. The pDDIs were further categorized according to their mechanism of action; pharmacodynamics (synergism or antagonism), pharmacokinetics (absorption, distribution, metabolism or elimination), other (mechanism known but other than pharmacokinetics & pharmacodynamics, eg - alterations in serum electrolytes, alterations in sedation etc.) and unknown [21]. The data was analyzed using Microsoft Excel and SPSS version - 21. Descriptive statistics and chi square test were used to describe data. Minimum sample size was calculated as 384 prescriptions using the following formula [22]:

$$ \mathrm{S}\mathrm{S} = {\mathrm{Z}}^2\mathrm{x}\ \left(\mathrm{P}\right)\ \mathrm{x}\ \left(1-\mathrm{P}\right)/{\mathrm{d}}^2 $$

SS: =Sample size for infinite population (where the population is greater than 50,000)
Z: = Z statistic for a level of confidence. For the level of confidence of 95 %, which is conventional, *Z* value is 1.96.
P: = Expected prevalence or proportion
d: = precision

The parameters used for calculating sample size were 95 % confidence level, an expected prevalence of 50 % and 5 % absolute precision.

## Results

Thousand prescriptions containing, 340 drugs (fifth level chemical substances) with a frequency of 3668 times from 198 classes (fourth level chemical sub-groups) were collected in four calendar months. All most all were hand written (99.8 %). Most prescriptions were from the private sector (73 %; 725/1000) and 15 % were from the government sector. Out of the total prescriptions 2.4 % were from a hospital ward, 12.5 % from an outpatient department, 27.7 % from a clinic and remaining were unclassified. Mean number of drugs per prescription was 3.95 (SD 2.2). The median and mode were 4.0 and 4.0 respectively. It had a positively skewed distribution.

### Most frequently prescribed substances

Atorvastatin, which is freely available in the universal free government health care system, was the most frequently prescribed drug (chemical substance) in both the government and the private and unclassified sector (Table 1). The most frequently prescribed chemical, pharmacological and therapeutic sub-groups are shown in Table 2. Twenty four different combination products were found and among them (*n* = 229) multivitamins (24 %), iron with folic acid (17 %) and calcium with vitamin D (14 %) were the most frequently prescribed (Table 3). From the chemical substances prescribed from the government sector 59 % (306/519) are available in the national list of essential medicines, Sri Lanka and in the private sector the figure is 50 % (1583/3149) [17].

**Table 1:** Top 10 drugs in prescriptions dispensed at SPC Anuradhapura 2015

Total		Government		Private & Unclassified	
Drug	% (*n* = 3668)	Drug	% (*n* = 519)	Drug	% (*n* = 3149)
Atorvastatin	4.0	Atorvastatin	7.1	Atorvastatin	3.5
Losartan	3.8	Losartan	7.1	Metformin	3.5
Metformin	3.6	Aspirin	6.5	Losartan	3.3
Pantoprazole	2.9	Metformin	4.2	Pantoprazole	2.9
Aspirin	2.7	Clopidogrel	3.3	Gliclazide	2.5
Gliclazide	2.5	Pantoprazole	2.9	Aspirin	2.1
Diclofenac sodium	1.8	Gliclazide	2.7	Diclofenac sodium	1.9
Celecoxib	1.6	Amlodipine	2.5	Celecoxib	1.7
Clopidogrel	1.6	Domperidone	2.5	Esomeprazole	1.6
Omeprazole	1.6	Omeprazole	2.1	Prednisolone	1.6

**Table 2:** Top 5 subgroups (*n* = 3668) in prescriptions dispensed at SPC Anuradhapura 2015

ATC 4th level	Percent	ATC 3rd level	Percent	ATC 2nd level	Percent
Chemical subgroup		Pharmacological subgroup		Therapeutic subgroup	
Proton pump inhibitor	7.0	Anti-inflammatory and anti-rheumatic products, non-steroids	8.8	Anti-inflammatory and anti-rheumatic products	8.8
HMG CoA reductase inhibitors	5.0	Blood glucose lowering drugs, excluding Insulins	8.3	Drugs used in diabetes	8.4
Angiotensin II antagonists, plain	4.9	Drugs for peptic ulcer and gastro-oesophageal reflux disease (GORD)	7.6	Drugs for acid related disorders	7.9
Platelet aggregation inhibitors excluding heparin	4.3	Lipid modifying agents, plain	5.8	Anti-bacterials for systemic use	7.3
Sulfonylureas	3.6	Angiotensin II antagonists, plain	4.9	Lipid modifying agents	5.8

**Table 3:** Combination products prescribed in prescriptions dispensed at SPC Anuradhapura 2015

No	Groups of combined preparations	% (*n* = 229)
01	Vitamins	36
02	Anti-anemic preparations	17
03	Mineral supplements	14
04	Drugs for obstructive airway diseases	11
05	Analgesics	09
06	Corticosteroids, dermatological preparations	04
07	Anti-acne preparations	04
08	Drugs for acid related disorders	02
09	Vasoprotectives	01
10	Antifungals for dermatological use	01
11	Anti-Parkinson drugs	01

### Completeness and legibility of prescriptions

Table 4 shows assessment of completeness of the prescriptions dispensed at SPC, Anuradhapura.

**Table 4:** Completeness of prescriptions (Total = 1000) dispensed at SPC Anuradhapura 2015

Component of the prescription	Present (complete/correct)
Patient information	
1. Name of patient	94 (42^c^)
2. Age of patient	79
3. Gender of patient	70
4. Address of patient	5
Prescriber information	
5. Name of prescriber	90 (98^c^)
6. Signature of prescriber	84
7. SLMC registration number	35
8. Place of prescribing	76
9. Contact details of prescriber	53
10. Qualifications of the prescriber	86
11. Prescriber's rubber stamp containing - Full name, qualifications, and registration number below his signature	89 (36^c^)
Drug information	
12. Route of administration	7 (90^b^)
13. Generic name of drug	16 (87^b^)
14. Dose of drug	93 (33^b^)
15. Frequency of drug	97 (68^b^)
16. Duration of drug	92 (84^b^)
Other information	
17. Treatment symbol	48 (61^b^)
18. Date of prescribing	88 (94^c^)
19. Diagnosis of the disease^a^	41
20. Refill information^a^	6

^a^Item 19 & 20 are in addition to the guidelines available in the BNF and WHO manual [3, 4]

^b^Correct, ^c^Complete

Address of the patient, SLMC registration number, route of administration, generic name, treatment symbol, diagnosis and refill information were seen in less than half of the prescriptions. Information for the package label was not seen in any of the prescriptions. Out of the prescriptions which had prescriber's rubber stamp, only 39 % (343/892) had the SLMC registration number. Omissions and/or errors in generic name, dose and SLMC registration number were respectively 89 %, 72 %, 62 % (*n* = 845) in the private sector and were significantly different when compared to 67 %, 54 %, 82 % (*n* = 155) in the government sector (Table 5). Most of the prescriptions were legible with effort (65 %). Legible and illegible prescriptions were 26 and 9 % respectively.

**Table 5:** Errors in prescriptions dispensed at SPC Anuradhapura 2015

	Route		Generic name		Dose		Frequency		Duration		pDDIs		Drug class Duplication		SLMC Number	
	(*n* = 1000)		(*n* = 1000)		(*n* = 1000)		(*n* = 1000)		(*n* = 1000)		(*n* = 887)		(*n* = 887)		(*n* = 1000)	
	C	I	C	I	C	I	C	I	C	I	P	A	P	A	C	I
Government	11	144	52	103	71	84	105	50	120	35	82	47	25	104	28	127
Private & Unclassified	52	793	93	752	233	612	558	287	649	196	384	374	105	653	320	525
*X* ^2^ (df = 1)	0.070		51.883		19.73		0.103		0.004		6.856		2.269		21.778	
*P*-value	0.792		0.000		0.000		0.748		0.950		0.009		0.132		0.000	

C – Present, Correct or Complete; I – absent, Incorrect or Incomplete, P - Present; A - Absent

### Drug duplication

Drug duplication was found in three prescriptions with omeprazole, etoricoxib and cetirizine. Drug class (chemical sub-group) duplication was found in 130 out of 887 prescriptions, which had two or more drugs, involving 19 classes. The commonest was non-steroidal anti-inflammatory drugs (NSAIDs) (43 %; 56/130). Chemical subgroup duplication doesn’t have a significant difference with regards to the sector (Table 5).

### Potential drug - drug interactions

Out of 887 prescriptions, which had two or more drugs, 466 (52.5 %) prescriptions had 1376 pDDI with a mean of 1.6 (SD 2.5) per prescription. It was a positively skewed distribution with median and mode being one and zero respectively. Out of them 94 (7 %) were serious, 1017 (74 %) significant and 265 (19 %) minor pDDIs. Table 6 shows the commonest pDDIs categorized according to the severity. In a single prescription the maximum numbers of serious, significant and minor pDDIs were 05, 15 and 04 respectively. The highest number of pDDI in a single prescription was 21 (this prescription had 14 drugs in it). Hundred and seventy eight different drugs were involved in pDDIs. The commonest pDDI was aspirin-losartan (4 %, *n* = 1376). Pharmacokinetics was the commonest mechanism involved in serious (40.4 %, *n* = 94) and minor (50.6 %, *n* = 265) pDDIs. Commonest mechanism involved in significant pDDI was ‘other’ (29.4 %, *n* = 1017). Additional file 2 shows commonest pDDIs for different mechanisms. pDDIs was low in the prescriptions originating from private sector (51 %; 384/758) compared to the government sector (64 %; 82/129) (Table 5).

**Table 6:** Common pDDIs of prescriptions dispensed at SPC Anuradhapura 2015

Combination (%)	Mechanism of pDDI according to the Medscape drug interaction checker [21]
Serious (Total = 94)	
Methotrexate-Leflunomide (6.4)	Leflunomide increases toxicity of methotrexate by pharmacodynamic synergism.
Meloxicam-Methotrexate (5.3)	Meloxicam increases levels of methotrexate by decreasing renal clearance.
Clopidogrel-Omeprazole (5.3)	Omeprazole decreases effects of clopidogrel by affecting hepatic enzyme CYP2C19 metabolism.
Clopidogrel-Esomeprazole (5.3)	Esomeprazole decreases effects of clopidogrel by affecting hepatic enzyme CYP2C19 metabolism.
Atorvastatin-Vitamin B3 (5.3)	Either increases toxicity of the other by pharmacodynamic synergism.
Atorvastatin-Fenofibrate (5.3)	Either increases effects of the other by pharmacodynamic synergism.
Significant (Total = 1017)	
Aspirin-Losartan (5.5)	Aspirin decreases effects of losartan by pharmacodynamic antagonism. losartan and aspirin both increase serum potassium.
Losartan-Frusemide (3.2)	Losartan increases and furosemide decreases serum potassium.
Aspirin-Clopidogrel (3.0)	Either increases toxicity of the other by pharmacodynamic synergism.
Clopidogrel-Pantoprazole (2.3)	Pantoprazole decreases effects of clopidogrel by affecting hepatic enzyme CYP2C19 metabolism.
Celecoxib-Diclofenac sodium (2.0)	Celecoxib and diclofenac both increase anticoagulation and serum potassium.
Aspirin-Frusemide (2.0)	Aspirin increases and furosemide decreases serum potassium.
Minor (Total = 265)	
Glipizide - Sitagliptin (7.6)	Either increases effects of the other by pharmacodynamic synergism.
Metformin - Vitamin B12 (5.7)	Metformin decreases levels of cyanocobalamin by unspecified interaction mechanism.
Metformin-Frusemide (3.8)	Metformin decreases levels of furosemide by unspecified interaction mechanism. Furosemide increases levels of metformin by unspecified interaction mechanism.
Methotrexate - Folic acid (3.4)	Folic acid decreases effects of methotrexate by pharmacodynamic antagonism.
Aspirin-Diltiazem (2.6)	Diltiazem increases effects of aspirin by unknown mechanism.
Metformin-Hydrochlorothiazide (2.6)	Hydrochlorothiazide will increase the level or effect of metformin by basic (cationic) drug competition for renal tubular clearance. Hydrochlorothiazide decreases effects of metformin by pharmacodynamic antagonism.

## Discussion

Statins and combination of aspirin-losartan was the most frequently prescribed and commonest pDDI respectively. Patient address, SLMC registration number, route, generic name, treatment symbol, diagnosis and refill information were absent in more than half of the prescriptions. Most prescriptions were legible with effort. Almost all prescriptions were handwritten. There were no prescriptions produced by electronic prescribing system. This may be because of lack of equipment, funding, overcrowding or lack of computer literacy. Concerns on consumer security, privacy and confidentiality would have contributed [23]. Previous studies have shown that the electronic prescribing system leads to reduction of prescription errors [18, 24].

There were 155 prescriptions from the government sector. Unavailability of the drugs, long queues at the government drug dispensing counters and patient preference may have led to the above finding. Out of the drugs prescribed from the government and private sectors, 59 % and 50 % respectively were from the national list of essential medicines [17]. A study conducted in Galle, Sri Lanka during the year 2002 had found 39.6 % (322/812) of the drugs prescribed in the private sector were from this national list [25]. This list of essential medicines was updated in 2014 and may have resulted in the change. The overall, rural and urban percentages of drugs prescribed from the essential drug list of a study in India were 37, 35 and 40 respectively [26]. Unclassified group of prescriptions with regards to sector may be due to prescriber’s negligence or non-institutional prescribing.

Average number of drugs per prescription was 3.95 with a median of four. In a previous study this number was six (median) among elderly patients in Sri Lanka [27]. In a study of 10 developing countries the average number of drugs per prescription was between 1.3 and 2.2 for general outpatient encounters [28]. This shows that number of drugs per prescription is high in Anuradhapura. Average number of drugs per prescription in rural and urban areas were 4 and 5 respectively in an Indian study [26]. Top three combination products (67 %; 154/229) dispensed are vitamins and minerals. Lack of knowledge on rational prescribing, differences in guidelines and placebo prescribing may be the reasons.

The US review showed antihypertensive and levothyroxine as the top chemical sub-group and chemical substance by prescriptions respectively [29]. The UK Report found amoxicillin as the top chemical substance prescribed [30]. Our study found overall proton pump inhibitors and atorvastatin as the top chemical sub-group and chemical substance by prescriptions respectively. They are also available in the government pharmacies. Antibiotics were the top chemical sub-group prescribed in a study conducted in rural India [26]. Differences in disease patterns and prescribing patterns would have contributed for the above finding.

SLMC registration number is given to all qualified doctors who practice and prescribe in Sri Lanka [31]. Using the SLMC registration number indirectly helps to stop quacks practicing medicine by identifying legitimate allopathic practioners. Only 35 % of the prescriptions had the number. Even among the prescriptions (*n* = 890) that had prescriber’s rubber stamp only 39 % had the SLMC registration number. Absence of SLMC registration number was more common in prescriptions originating from government sector. A previous study in Kandy, Sri Lanka found SLMC registration number only in 16 % (*n* = 200) [32]. Prescribers from private sector may be more concerned about their right to prescribe and to identify themselves as western medical practitioners, hence increased use of SLMC registration number that identifies themselves as prescribers with provenance to practice allopathic medicine.

Generic name was present only in 11 % of the private sector prescriptions which was lesser than in the study conducted in 2002 at the private sector of Galle, Sri Lanka (36.7 %) [25]. The drugs prescribed by generic name were 52 and 71 % respectively in rural and urban India [26].

Only 48 % of the prescriptions had the treatment symbol. The treatment symbol is derived from *Recipe* (Latin for ‘take’) and gives a legal validity to prescriptions. After the symbol the prescriber should write the name and strength of the drug [4].

Name, age, gender and diagnosis were missing in more prescriptions (6 %, 21 %, 30 %, 59 %) of rural Sri Lanka compared to rural India (0 %, 0 %, 0 %, 28 %). The prescribers signature was present in more prescriptions of rural Sri Lanka (84 %) compared to rural India (45 %).

The WHO manual and the BNF [3, 4] has not included diagnosis and refill information however it may be important for following reasons. One drug can be prescribed for two different reasons (Eg-Propanolol in hypertension and migraine). Mentioning of diagnosis reduces confusion among the drug dispensers. Refill information (Eg - Do not repeat/Repeat once) will help curtail drug abuse and misuse. Diagnosis (41 %) and refill information (6 %) were seen in less than half of the prescriptions.

According to the WHO manual it is the legal duty of the doctor to write legibly [4]. In our study 26 % of the prescriptions are legible and 9 % are illegible. In Galle 26 % (*n* = 812) [25] and in Kandy 50 % (*n* = 200) [32] were illegible. However methods used to assess legibility in Kandy is not clear as it is an abstract. Legibility was 23 and 59 % in rural and urban areas of India respectively [26]. However the method used to assess legibility was not clear. Compared to a study done in Saudi Arabia (1 %) [33], drug duplication was lower in our study (0.3 %).

Consideration of pDDIs in a prescription not only helps to avoid or minimize them it also assists in monitoring and warning the patient on pDDIs. Comparison of the findings of pDDIs was made with the study done in Ahmedabad, India in 2014 [34] again using the Medscape drug interaction checker (Additional file 2). The comparison shows similarity in distribution of severity of pDDIs and commonest pharmacodynamic pDDI (aspirin-losartan). Percentage of prescriptions having pDDIs and mean pDDI per prescription was low in our study (53 % &1.6) compared to the Indian (83 % & 5.9). Maximum number of pDDI per prescription was 21 in our study compared to 33 in the Indian. The commonest pDDI was aspirin-losartan in our study and metoprolol-aspirin in the Indian [34]. The reasons for the dissimilarity may be due to the differences in the disease and prescribing pattern.

Though we used the open access Medscape drug interaction checker there are formulas and commercially available drug information systems [9]. The commonest combination causing pDDIs was aspirin-losartan but a study done using the Micromedex system in a cardiac clinic at south-west Ethiopia found enalapril-frusemide as the commonest [35]. The reason may again be the differences in the epidemiology of disease and prescribing pattern. pDDIs have a significant difference with regards to the sector (*p* = 0.009). pDDIs was low in the prescriptions originating from private sector compared to the government sector. Reasons may be lack of resource to check for pDDI, lack of time to check for pDDI due to overcrowding and lack of concern.

The study had limitations such as being confined to SPC, Anuradhapura, unavailability of a standard prescription format in Sri Lanka and the pDDIs being assessed using only open access software. Yet the present study was capable in producing valuable findings which could provide a basic platform for future studies.

## Conclusion

Fifteen percent of the prescriptions originate from government sector. Reasons that drive consumers away from the government drug dispensing services (where products and services are free) are not clear. SLMC registration number and trade names were seen more in prescriptions originating from the private sector. Findings on legibility, completeness, drug duplication and pDDIs show that there is room for improvement in prescriptions originating from rural Sri Lanka. Island wide studies (to find out generalized data), root cause analysis (on prescription errors) and formation of a validated prescription template (in view of regulating prescription writing) are recommended.

## Abbreviations

ATC, anatomical therapeutic and chemical; BNF, British National Formulary; GDP, gross domestic product; pDDI, potential drug-drug interaction; MBBS, Bachelor of Medicine, Bachelor of Surgery; NSAIDs, non steroidal anti inflammatory drugs; SD, standard deviation; SLMC, Sri Lanka Medical Council; SPC, State Pharmaceutical Corporation; SPSS, statistical package for the social science; UK, United Kingdom; US, United States; WHO, World Health Organization

## Additional files

Additional file 1: Assessment of legibility and completeness of prescriptions received at State Pharmaceutical Corporation, Anuradhapura-Check List. (DOC 53 kb) Additional file 2: Comparison of findings on pDDIs found by Medscape drug interaction checker. (DOC 39 kb)
