# Supplementary material for: Drug utilization, prescription errors and potential drug-drug interactions: an experience in rural Sri Lanka
Source: BMC Pharmacol Toxicol. 2016 Jun 25;17:27. doi: 10.1186/s40360-016-0071-z (PMC4921016; doi:10.1186/s40360-016-0071-z)
Supplement: Additional file 1: — Assessment of legibility and completeness of prescriptions received at State Pharmaceutical Corporation, Anuradhapura-Check List. (DOC 53 kb) [file 40360_2016_71_MOESM1_ESM.doc]

| **Additional file-1 Assessment of legibility and completeness of prescriptions received at**  **State Pharmaceutical Cooperation, Anuradhapura – Check List**  1.0 ID No: ___ ___ ___ ___  2.0 Type of prescription | | |
| --- | --- | --- |
| Hand written prescription |  | |
| Printed prescription |  | |
| 3.0 Sector | | |
| Government sector |  | |
| Private sector |  | |
| Unclassified |  | |
| 4.0 Unit | | |
| Ward |  | |
| Clinic |  | |
| Out Patient Department (OPD) |  | |
| Unclassified |  | |
| 5.0 Likert scale to assess legibility | | |
| 1 - Illegible |  | |
| 2 - Legible with effort |  | |
| 3 - Legible |  | |
| 6.0 Check list to assess completeness | Present | Correct/Complete |
| Patient information | | |
| 1. Name of patient |  |  |
| 1. Age of patient |  |  |
| 1. Gender of patient |  |  |
| 1. Address of patient |  |  |
| Prescriber information | | |
| 1. Name of prescriber |  |  |
| 1. Signature of prescriber |  |  |
| 1. SLMC registration number |  |  |
| 1. Place of prescribing |  |  |
| 1. Contact details of prescriber |  |  |
| 1. Qualifications of the prescriber |  |  |
| 1. Prescriber's rubber stamp containing - Full name, qualifications, and registration number below his signature |  |  |
| Drug information | | |
| 1. Route of administration |  |  |
| 1. Generic name of drug |  |  |
| 1. Dose of drug |  |  |
| 1. Frequency of drug |  |  |
| 1. Duration of drug |  |  |
| 1. Information for the package label |  |  |
| Other information | | |
| 1. Treatment symbol |  |  |
| 1. Date of prescribing |  |  |
| 1. Diagnosis of the disease |  |  |
| 1. Refill information |  |  |

| **Additional file-2 Comparison of findings on pDDIs found by Medscape drug interaction checker** | | |
| --- | --- | --- |
| **Features** | **Anuradhapura, 2015** | **Ahmedabad, 2014** |
| Site | SPC, Anuradhapura, Sri Lanka | Medicine, Outpatient Department of a tertiary teaching care hospital, Ahmedabad, India |
| Total No. of pDDIs | 1376 | 2066 |
| No. Prescriptions having pDDIs | 466 out of 887 (53%) | 292 out of 350 (83%) |
| Mean of pDDIs | 1.6 (SD 2.5) | 5.9 (SD 6.0) |
| No. of serious pDDIs | 94/1376 (7%) | 76/2066 (4%) |
| No. of significant pDDIs | 1017/1376 (74%) | 1516/2066 (73%) |
| No. of minor pDDIs | 265/1376 (19%) | 474/2066 (23%) |
| Maximum number of pDDI per prescription | 21 | 33 |
| Most No. of serious pDDIs | MTX - Leflunomide 6/94 (6.4%) | proton pump inhibitors-antiplatelet 26/76 (34%) |
| Most common pDDI | Aspirin - Losartan 56/1376 (4%) | Metoprolol - Aspirin 126/2066 (6%) |
| No. of pharmacodynamics pDDIs | 280/1376 (20%) | 1424/2066 (69%) |
| No. of pharmacokinetic pDDIs | 281/1376 (20%) | 553/2066 (27%) |
| No. of unknown, other & dual pDDIs | 815/1376 (60%) | 89/2066 (4%) |
| Commonest Pharmacodynamic pDDI | Aspirin - Losartan* 56/280 (20%) | Aspirin - Losartan 123/1424 (9%) |
| Commonest Absorption pDDI | Pantoprazole - Vitamin B1 26/50 (52%) | Aspirin - Vitamin B1 52/217 (24%) |
| Commonest Distribution pDDI | Nil | Aspirin – Glimepiride 26/32 (81%) |
| Commonest Metabolism pDDI | Clopidogrel - Pantoprazole 23/166 (14%) | Clopidogrel - Rabeprazole 23/145 (16%) |
| Commonest Elimination pDDI | Metformin-Hydrochlorothiazide 7/65 (11%) | Aspirin - Hydrochlorothiazide 14/106 (13%) |
| Commonest Unknown pDDI | Losartan - Frusemide 32/304 (11%) | Aspirin - Glimepride 26/89 (29%) |

*Shows dual mechanism
