# Supplementary material for: Drug utilization, prescription errors and potential drug-drug interactions: an experience in rural Sri Lanka
Source: BMC Pharmacol Toxicol. 2016 Jun 25;17:27. doi: 10.1186/s40360-016-0071-z (PMC4921016; doi:10.1186/s40360-016-0071-z)
Supplement: Additional file 2: — Comparison of findings on pDDIs found by Medscape drug interaction checker. (DOC 39 kb) [file 40360_2016_71_MOESM2_ESM.doc]

| **Additional file-2 Comparison of findings on pDDIs found by Medscape drug interaction checker** | | |
| --- | --- | --- |
| **Features** | **Anuradhapura, 2015** | **Ahmedabad, 2014** |
| Site | SPC, Anuradhapura, Sri Lanka | Medicine, Outpatient Department of a tertiary teaching care hospital, Ahmedabad, India |
| Total No. of pDDIs | 1376 | 2066 |
| No. Prescriptions having pDDIs | 466 out of 887 (53%) | 292 out of 350 (83%) |
| Mean of pDDIs | 1.6 (SD 2.5) | 5.9 (SD 6.0) |
| No. of serious pDDIs | 94/1376 (7%) | 76/2066 (4%) |
| No. of significant pDDIs | 1017/1376 (74%) | 1516/2066 (73%) |
| No. of minor pDDIs | 265/1376 (19%) | 474/2066 (23%) |
| Maximum number of pDDI per prescription | 21 | 33 |
| Most No. of serious pDDIs | MTX - Leflunomide 6/94 (6.4%) | proton pump inhibitors-antiplatelet 26/76 (34%) |
| Most common pDDI | Aspirin - Losartan 56/1376 (4%) | Metoprolol - Aspirin 126/2066 (6%) |
| No. of pharmacodynamics pDDIs | 280/1376 (20%) | 1424/2066 (69%) |
| No. of pharmacokinetic pDDIs | 281/1376 (20%) | 553/2066 (27%) |
| No. of unknown, other & dual pDDIs | 815/1376 (60%) | 89/2066 (4%) |
| Commonest Pharmacodynamic pDDI | Aspirin - Losartan* 56/280 (20%) | Aspirin - Losartan 123/1424 (9%) |
| Commonest Absorption pDDI | Pantoprazole - Vitamin B1 26/50 (52%) | Aspirin - Vitamin B1 52/217 (24%) |
| Commonest Distribution pDDI | Nil | Aspirin – Glimepiride 26/32 (81%) |
| Commonest Metabolism pDDI | Clopidogrel - Pantoprazole 23/166 (14%) | Clopidogrel - Rabeprazole 23/145 (16%) |
| Commonest Elimination pDDI | Metformin-Hydrochlorothiazide 7/65 (11%) | Aspirin - Hydrochlorothiazide 14/106 (13%) |
| Commonest Unknown pDDI | Losartan - Frusemide 32/304 (11%) | Aspirin - Glimepride 26/89 (29%) |

*Shows dual mechanism
